# Genome-Wide Analyses of Recombination Prone Regions Predict Role of DNA Structural Motif in Recombination

**DOI:** 10.1371/journal.pone.0004399

**Published:** 2009-02-09

**Authors:** Prithvi Mani, Vinod Kumar Yadav, Swapan Kumar Das, Shantanu Chowdhury

**Affiliations:** 1 G. N. Ramachandran Knowledge Centre for Genome Informatics, Institute of Genomics and Integrative Biology, CSIR, Delhi, India; 2 Proteomics and Structural Biology Unit, Institute of Genomics and Integrative Biology, CSIR, Delhi, India; 3 Functional Genomics Unit, Institute of Genomics and Integrative Biology, CSIR, Delhi, India; Institute of Infectious Disease and Molecular Medicine, South Africa

## Abstract

HapMap findings reveal surprisingly asymmetric distribution of recombinogenic regions. Short recombinogenic regions (hotspots) are interspersed between large relatively non-recombinogenic regions. This raises the interesting possibility of DNA sequence and/or other *cis*- elements as determinants of recombination. We hypothesized the involvement of non-canonical sequences that can result in local non-B DNA structures and tested this using the G-quadruplex DNA as a model. G-quadruplex or G4 DNA is a unique form of four-stranded non-B DNA structure that engages certain G-rich sequences, presence of such motifs has been noted within telomeres. In support of this hypothesis, genome-wide computational analyses presented here reveal enrichment of potential G4 (PG4) DNA forming sequences within 25618 human hotspots relative to 9290 coldspots (p<0.0001). Furthermore, co-occurrence of PG4 DNA within several short sequence elements that are associated with recombinogenic regions was found to be significantly more than randomly expected. Interestingly, analyses of more than 50 DNA binding factors revealed that co-occurrence of PG4 DNA with target DNA binding sites of transcription factors c-Rel, NF-kappa B (p50 and p65) and Evi-1 was significantly enriched in recombination-prone regions. These observations support involvement of G4 DNA in recombination, predicting a functional model that is consistent with duplex-strand separation induced by formation of G4 motifs in supercoiled DNA and/or when assisted by other cellular factors.

## Introduction

DNA in its double-stranded form (B-DNA conformation) is a critical genetic component for most organisms. Therefore, it is central to cellular function that strict control over integrity of the large and complex DNA molecule is maintained under constant challenge from a variety of factors. These include not only external environmental abuse (like chemicals and radiation) that lead to mutagenesis but also changes that are inherent to the double-stranded DNA form itself. Indeed, several forms of non-B DNA structural conformations resulting from repeat sequences have been demonstrated to be mutagenic and cause hereditary disorders (reviewed in [1], [2]). Several different non-B DNA forms have been reported to have functional consequence; these include cruciforms, triplexes, slipped structures, G-quadruplex, left-handed Z DNA and bent DNA [3]–[5]. It is believed that formation of non-B DNA conformations (inherently of high energy states) is supported by duplex destabilization resulting from negative supercoiling induced by multiple cellular processes including transcription, replication and also protein binding. Apart from this, in specific instances non-B DNA conformations have been mechanistically linked to recombination-related events [6], [7]. A particular form – the G-quadruplex DNA has seen a resurgence of interest due to interesting findings directly relating such structures to gene regulation in prokaryotes [8] and eurkaryotes [9], [10]. However, though implicated in literature the G-quadruplex DNA has not been directly studied in the context of recombination. Herein, we have addressed the question of involvement of non-B DNA conformation in recombination in a genome-wide scale using G-quadruplex DNA.

**Figure 1:**
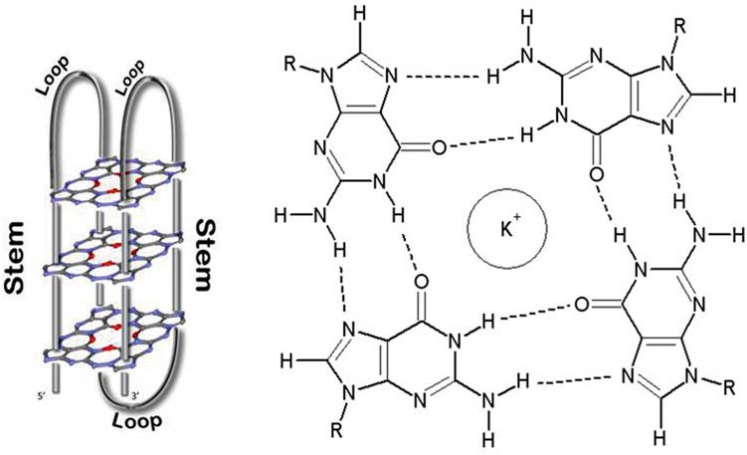
(a) G-quadruplex or G4 DNA. The stem and loop form of the G4 DNA is shown. Stem is composed of 3 planar structures of intermolecular H bonded guanines. (b) Wireframe representation of a tetrad stabilized by single K^+^ where the H-bonds are shown by broken lines.

A particular arrangement of guanine-rich sequences adopts unique four-stranded conformations known as G-quadruplex or G4 DNA (Figure 1(a)) [11]–[13]. Hydrogen-bonded self-assembly of four guanine bases can form planar arrangements called tetrads or G-quartets (Figure 1(b)), where charge coordination by monovalent cations (especially K^+^) stabilize stacking of G-quartets resulting in intramolecular or intermolecular association of four DNA strands in parallel or antiparallel orientation (for review, see [14]–[17]). Though *in vitro* G4 DNA formation is known for several decades [18], biological relevance of this alternate or non-B DNA form has received more attention in recent years due to several interesting findings. G4 DNA was found as repeated motifs present in telomere ends of several species [19], its role in telomere maintenance was discovered [20] and more recently shown to form *in vivo* in a cell-cycle dependent fashion [21]. Recently prevalence of sequence with potential to form G4 DNA was noted outside telomeres across the whole human genome [22], particularly within promoters in human [9], [23] and other mammals [10], chicken [24] and several bacteria [8] suggesting the possibility of G4 DNA as a *cis*-regulatory site. These findings support results showing regulatory role of the G4 DNA present in promoter of the oncogene *c-MYC* [25] and *PDGF-A* [26]. Apart from this, chromosomal regions containing guanine-rich sequence which are capable of forming G4 DNA include immunoglobin heavy chain switch regions [27], G-rich minisatellites [28], [29] and rDNA [30].

The dynamic folding/unfolding character of the G4 DNA structure is of interest in the context of its role as a *cis*-regulatory site. Several studies have addressed these aspects *in vitro* in single-stranded forms [31] as well as in the presence of competing hybridization from the complementary strand, a situation more likely within genomes [32]. This possibility of inducing strand separation due to formation of a G4 DNA could be of functional relevance in processes other than transcription. Single stranded DNA transiently formed during meiosis within the synaptonemal complex could result in G4 DNA from G-rich sequences [33]. Indeed, several factors required for recombination have been found to bind G4 DNA. Hop1, a meiosis specific protein in *Saccharomyces cerevisiae* promotes formation of G4 DNA by preferentially binding to such motifs [34]. Recent studies show Mre11, Rad50 and Xrs2, which are subunits of the MRX complex formed during meiotic recombination in *S. cerevisiae* have high affinity to the G-quadruplex structures [35], [36]. On the other hand, genome-wide studies using large scale single nucleotide polymorphism data across multiple populations show surprisingly asymmetric distribution of recombinogenic regions, which are distributed as relatively short regions with higher recombination propensity (hotspots) interspersed between large relatively non-recombinogenic regions [37], [38]. Moreover, similar primary sequence composition shows different recombination propensity as observed in a comparative analysis of human and chimpanzee [39], raising the question of particular short sequences and other genomics features that could influence recombination propensity. Comparative studies conducted earlier yielded several short sequence features that were enriched within hotspots [40]. However, presence of non canonical sequences that can result in local non-B DNA structures like, G4 DNA has not been studied yet.

Here our goal was to first analyze the presence of sequences that could potentially adopt G4 DNA structures with the reasoning that any functional role would be reflected in occurrence within hotspots that is significantly different from regions with low recombination propensity. Using computational methods, sequence patterns with potential to form G4 DNA were mapped within 32996 hotspots and corresponding flanking regions in human. We found that potential G4 (PG4) DNA forming sequences were significantly enriched within hotspots, while a control sequence that would not adopt structure was not differentially distributed. We also tested and found that within a 50 bp distance PG4 DNA co-occurrence with previously reported short sequence motifs (with enriched presence in hotspots) was significantly higher than expected randomly. Interestingly, testing the association of PG4 DNA with >50 transcription factor binding sites (TFBS) within the hotspots revealed significant co-occurrence with target sites for three factors – c-Rel, NF-kappa B (p50&p65) and Evi-1. Based on these findings, we propose G4 DNA could be one of the determinants of recombination wherein the single-stranded fold back structure could assist in strand separation and homologous pairing.

## Results and Discussion

**Figure 2:**
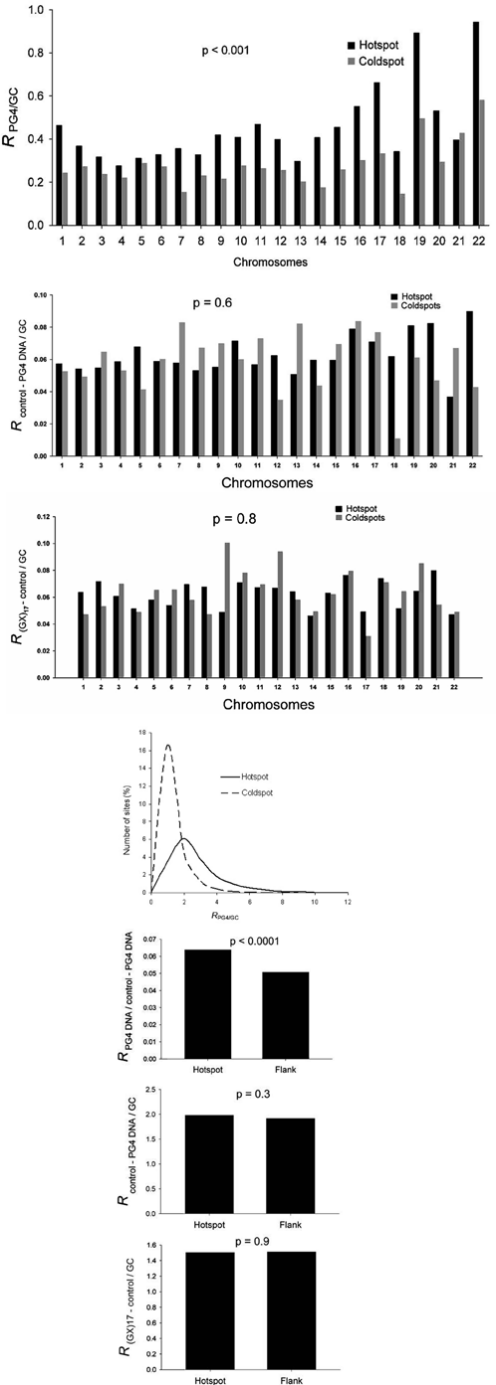
Recombinogenic regions (hotspots) are enriched in PG4 DNA. (a) Average *R*_PG4/GC_ (PG4 DNA density normalized to the GC frequency for each region) in 25618 hotspots and 9290 coldspots reported by Myers *et al* (29). (b,c) Analysis of control motifs: average density (normalized for GC content) of the control-PG4 DNA motif (b) and the (GX)_17_-control (c) in 25618 hotspots and 9290 coldspots. (d–f) Analysis of hotspots from HapMap: frequency plot of *R*_PG4/GC_ within hotspots and coldspots (d); average density of PG4 DNA normalized to density of the control-PG4 DNA (e), average density of control-PG4 DNA normalized for GC-frequency (f) and average density of (GX)_17_-control normalized for GC-frequency (g) compared for hotspots and respective flanking regions.

We searched for PG4 DNA (with three to five guanine bases in stem and one to seven bases in loop (Methods and Figure 1)) within 25618 hotspots (∼303 Mb) and 9290 coldspots (∼37 Mb) that were reported by Myers et al. [40]. In this study genome wide recombinogenic regions were reported after studying 1,586,383 single nucleotide polymorphisms (SNP) across 71 American individuals comprising three different population groups. In order to test for any overall difference between PG4 DNA content in hotspots versus coldspots, we estimated the density of PG4 DNA in each reported region individually and used this to calculate average PG4 density in hotspots and coldspots in a chromosome-wise fashion. Considering the G-rich nature of the sequence, PG4 density was normalized for GC content (which was noted to be significantly different in hotspots versus coldspots (Methods)) and expressed as *R*_PG4/GC_ (Methods). Average *R*_PG4/GC_ was more within hotspots across all chromosomes (0.40 in hotspots compared to 0.25 in coldspots; p = 2.75×10^−277^, z = −35.5; Figure 2(a)). On considering individual sites we noted about 37% of the hotspots (9517/25618) harbored at least one PG4 site while it was 13.8% (1285/9290) in case of coldspots.

In the above analyses, average PG4 density was considered (thereby taking into account length of the sites). However, it is not clear whether recombination propensity is influenced by length of the site and/or frequency of SNPs found within any particular region. To rule out this possibility, we used a set of 9290 hotspots and corresponding coldspots that were matched for length and SNP density [40]. PG4 density was calculated after normalizing for GC content. *R*_PG4/GC_ in this case was significantly higher in hotspots (0.310 and 0.256 for hotspots and coldspots respectively; p = 1.6×10^−6^, z = −4.8; Table S1). Analysis of the (−) strand using the matched hotspots and coldspots also revealed higher *R*_PG4/GC_ within hotspots relative to coldspots with almost similar densities as the (+) strand (0.304 and 0.251 for hotspots and coldspots, respectively; p = 1.2×10^−8^, z = −5.7). Furthermore, to test whether the enrichment was due to the structural motif and not sequence we devised two control sequences: (a) control-PG4 DNA and (b) (GX)_17_-control that are unlikely to adopt the G4 DNA structure (details given in Methods). Occurrence of the controls was analyzed as done for PG4 DNA. Average *R*_control-motif/GC_ (*i.e.*, density of the control motifs normalized for GC content) was calculated for both controls in all the 25618 hotspots and 9290 coldspots and found to vary without significant difference across all chromosomes (p = 0.6 and 0.8 for control-PG4 and the (GX)_17_-control, respectively; Figure 2(b) and 2(c)). The average *R*_control-motif/GC_ did not significantly vary on considering the 9290 hotspots and coldspots matched for length and SNP density (Table S1).

Next, we used another independent genome-wide dataset reported by HapMap which estimated recombination rate after studying 3,107,620 SNPs in 269 individuals from four different populations across the world [41]. Enrichment of PG4 DNA within hotspots reported in HapMap was tested relative to the region immediately flanking each hotspot (which are usually of lower average recombination rate [38]). This comprised of 32996 hotspots in 22 human chromosomes spanning 181 Mb of sequence. Our approach in this case allowed us to analyze corresponding regions of relatively low recombinogenic potential for each hotspot across all chromosomes. Out of 32996 sites 8984 regions (27.2%) harbored one or more PG4 motifs; *R*_PG4/GC_ within the 8984 sites was about 2.4-fold higher than the corresponding flanking regions (*R*_PG4/GC_ = 1.67 in hotspots versus 0.68 within flanking regions, p<1.15×10^−299^, z = −61.9, Wilcoxon Signed Ranks Test for two related samples).

In order to further confirm these findings, we selected hotspots and coldspots from the HapMap data using the recombinogenicity index cM/Mb (average of four different populations). We considered regions with cM/Mb ≥10 as hotspots and regions with cM/Mb≤0.1 as coldspots. Based on this criterion, 25287 hotspots and 83893 coldspots were identified. Out of these, 4521 (17.9%) hotspots and 19550 (23.3%) coldspots were found with one or more PG4 motifs. We considered the regions having at least one/more PG4 motifs and noted a remarkable enrichment in average *R*_PG4/GC_ within hotspots relative to coldspots (2.51 versus 0.96 for hotspots and coldspots, respectively; p<1.15×10^−299^, z = −69.2, Mann Whitney U test). Figure 2(d) shows the frequency plot of the 4521 hotspots and 19550 coldspots using *R*_PG4/GC_ of individual sites indicating the enrichment of PG4 motif density within the more recombinogenic sites. Therefore, from the analysis using recombinogenicity index it appears that enrichment (or presence of multiple PG4 motifs) rather than single or isolated presence of motifs is required for higher recombination. PG4 density was expressed as a ratio of the control-PG4 density within each hotspot and corresponding flanking region in order to correct using the simulated control sequence (*R*_PG4/control-PG4_; Figure 2(e)). *R*_PG4/control-PG4_ was significantly enriched within hotspots compared to corresponding flanking regions indicating relevance of the sequence with structure forming potential within recombinogenic regions (p = 1.4×10^−14^, z = −7.7). We also tested density of the control-PG4 DNA and (GX)_17_-control within all the flanking regions and compared this with the occurrence density within hotspots after regressing GC content (*R*_control/GC_ as done for PG4). Occurrence of both controls within flanking regions was not significantly different from hotspots (p = 0.3 and 0.9 for control-PG4 and (GX)_17_-control, respectively; Figure 2(f) and 2(g)).

It is generally perceived that recombination requires open chromatin devoid of nucleosomes where *trans*-factors required for recombination find access to DNA sequence. Keeping this in mind, we reasoned that DNase I hypersensitive sites (DHS) that generally constitute open chromatin may show further enrichment of PG4 DNA within hotspots relative to coldspots. The generalization about DHS being nucleosome-free is with the caveat that there are several examples where DHS are known to harbor nucleosomes that could constitute an alternative form of histone-DNA complex (reviewed in reference [42]). We used the experimentally determined DHS sites reported by Sabo *et al.* in human B lymphoblastoid cells using a tiling array based method, where 2690 DHS were mapped into 1% of the human genome (ENCODE regions) [43]. Out of these, 271 DHS (∼72 kb) were found within 255 hotspots present in the ENCODE region while 33 DHS (∼8 kb) mapped into the 99 coldspots present within the ENCODE regions. Therefore, as expected there was ∼3-fold more DHS within hotspots (271/255 in hotspots versus 33/99 in coldspots) largely supporting the view that recombinogenic regions have more open chromatin (exclude nucleosomes). In order to test density of PG4 DNA within DHS in hotspots versus coldspots we determined *R*_PG4/GC_ of the DHS found in respective regions. No PG4 DNA could be identified within the 33 DHS present in coldspots. The average *R*_PG4/GC_ within 271 DHS present in hotspots was 0.9723. Interestingly, >2-fold enrichment of PG4 density was observed in DHS relative to the overall average *R*_PG4/GC_ (0.401) observed within 25618 hotspots. We further noted that 74 sites (2.7%) of the 2690 DHS harbored one or more PG4 DNA motifs. On considering the 271 DHS that were present within hotspots about 12 (4.4%) had one or more PG4 DNA motifs; the average *R*_PG4/GC_ within these 12 DHS was 21.95 (>50-fold enrichment over the genome-wide hotspot average). Though substantial enrichment of PG4 density was observed within the DHS in hotspots (12/271) this was statistically not significant when compared to the absence of PG4 motifs in 33 DHS occurring within coldspots (p = 0.37, Fisher's Exact test). Therefore, taken together this indicated enrichment of PG4 DNA within DHS present in hotspots and is in line with observations made earlier showing enriched PG4 DNA presence within DHS throughout the human genome [23]. However, the contribution of PG4 motifs in this context was not clearly discernible. One possible reason could be the relatively small number of DHS within hotspots/coldspots (present in the ENCODE regions) that were found to have PG4 motifs.

Meiotic crossovers events engage nucleoprotein assemblies known as synaptonemal complex for anchoring DNA strands at close proximity, wherein the presence of single stranded DNA has been postulated to result in G4 DNA formation [33]. This was supported by demonstrated effect of yeast meiosis-specific protein Hop1 in promoting synapses by binding to G4 DNA directly [34], [44]. In line with involvement of G4 DNA in synapses, recent results show that several meiosis-specific proteins in the MRX complex (Mre11, Xrs2 and Rad50) that induces synapses formation, directly binds to G4 DNA. Interestingly, though all three factors in the MRX complex independently bind G4 DNA, the affinity for G4 DNA increases when all three are in complex [36]. These results, particularly the Hop1-G4 DNA interactions was known for decades and therefore implications of G4 DNA and its role in recombination has been proposed for a long time. To the best of our knowledge, we have tested this hypothesis for the first time in a genome-wide context and found strong support for the role of G4 DNA in recombination. This is indicated by the presence of genomic features that are consistent with the presence of structural G4 DNA forms, but are unlikely to be due to primary sequence representing a G4 DNA.

Keeping in mind the enrichment of PG4 DNA within hotspots, we conjectured that PG4 DNA could be functionally associated with other known sequences elements found to be associated with hotspots. To test this, we selected 7 short enriched sequences (SES) (ranging from 6-mer to 9-mer, sequence is given in Methods), which top the list of short sequences that were found to be enriched in 32996 hotspots with respect to control regions of low recombination [40]. The 7 SES selected by us were also found to be enriched within hotspots found in human imprinted chromosomal regions in a recent study [45]. Furthermore, the SES, CCTCCCT and CCACGTGG have been noted to be enriched within meiotic recombination hotspots in another study [46]. We arbitrarily selected a window of 50 bp flanking the PG4 DNA for testing co-occurrence with an SES. Co-occurrences within this window was found for all SES in 32996 hotspots and significance was analyzed based on randomly expected number of co-occurrences given the individual frequency of each SES and PG4 DNA within a hotspot (see Methods for details of the significance analysis, which was based on a method published earlier [47]). This approach effectively rules out the possibility of observing higher co-occurrence in hotspots due to mere higher frequency of PG4 DNA/marker sequence because expected co-occurrence is calculated based on frequency of individual components in hotspot for each region. More than 100 co-occurrences were observed for three SES and are shown in Table 1 along with chi-square values obtained for significance. Co-occurrence observed with other SES were lower than 100 (Table S2). Number of co-occurrence for TACTGTTC was only 15; this appears to be consistent with a previous study, which also found TACTGTTC does not show enriched presence within hotspots occurring in human imprinted chromosomal regions [45]. Highest number of associations was observed for GGGGGT (4221), however, we noticed that this SES closely resembled a PG4 DNA sequence and hence was most likely to adopt a PG4 DNA structure with appropriate flanking sequence. Therefore, in order to check the validity these results, control analyses were done using the two sequences restricted for PG4 DNA formation. Number of co-occurrence of the control-PG4 DNA and (GX)_17_-control with each of the three SES (having more than 100 co-occurrences) is given in Table 1. For GGGGGT and CCTCCCT, chi-square obtained for the control motifs were at least an order lower than that observed for PG4 DNA. Chi-square for CCTCCCTG with control-PG4 DNA was also 4-fold lower than that observed for PG4 DNA. Though low, the chi-square observed for these motifs with control sequences was not insignificant. A possible reason for this could be the G-rich nature of the control sequences which renders their co-occurrence with G/C-rich SES within regions with high overall GC-content significant. CCTCCCT, which is present within the long terminal repeats of retrovirus-like retrotransposon THE1B (overrepresented within hotspots) showed the strongest signal for enrichment within hotspots [40]. Taken together, PG4 DNA co-occurrence with SES is independently significant in at least three cases but must be considered with the caveat that some of these co-occurrences appear significant in controls also and therefore may or may not be associated directly to structure. Nevertheless, these observations build a case for testing association of SES with PG4 motifs experimentally.

**Table 1:** PG4 DNA association with short enriched sequences (SES)

short enriched sequences (SES)	Observed number of co-occurrence		
	Significance (Chi-square$ of co-occurrence) is in parentheses		
	PG4 DNA	Control-PG4 DNA	(GX)17-control
**GGGGGT**	4221	37	58
	(7638524)	(12956)	(27452)
**CCTCCCT**	237	18	12
	(51342)	(3993)	(4021)
**CCTCCCTG**	139	14	4
	(37604)	(8576)	(1342)

$ Significance analysis in each case was done based on randomly expected co-occurrence (Methods).

**Figure 3:**
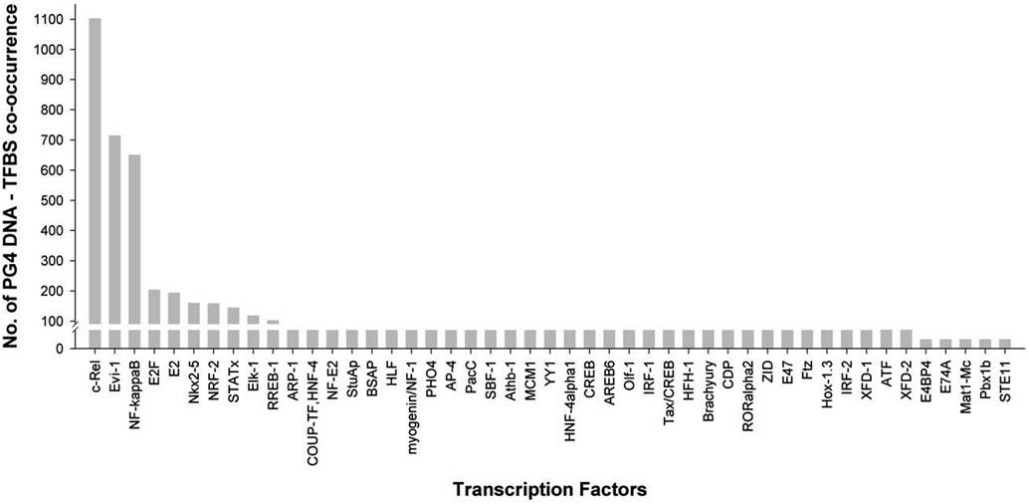
NF-kappa B, c-Rel and Evi-1 binding sites co-occur with PG4 DNA within hotspots. Co-occurrence within 50 bp of PG4 DNA with transcription factor binding sites of 49 factors (with one or more associations) in 32996 hotspots is shown.

Putative α hotspots in the human genome have been recently proposed, which exclusively depend on transcription factors for their activation [48]. Role of transcription factors in recombination has been observed in *S. cerevisiae* and *Schizosaccharomyces pombe* also [49], [50]. Rap1p binding to HIS4 loci in *S. cerevisiae* induces hotspot activity [49]. This was demonstrated by introducing substitutions/mutations in the Rap1p binding site upstream of *HIS4*, which lead to lower recombination propensity. Interestingly, Rap1p also induces G4 DNA formation [51]. Our current observation of PG4 DNA enrichment within hotspots, taken along with earlier studies showing G4 DNA interaction with factors like, Rap1p, Hop1 and components of the MRX complex in *S. cerevisiae* prompted us to ask if PG4 DNA was associated with any DNA binding factor(s) within hotspots. In order to test this, we analyzed presence of transcription factor binding sites (TFBS) within 50 bases of all PG4 DNA observed in 32996 hotspots. This was done for all TFBS present in TRANSFAC using the P-Match program [52] (Methods). TFBS for 59 transcription factors were observed within hotspots. Presence of a PG4 DNA site within 50 bp was noted for 47 factors, out of which 10 factors had more than 100 co-occurrences (Figure 3). Significance of co-occurrence was estimated as described previously [47] and is given in Methods. Co-occurrence was significant in all 10 cases (Table S3). More than 500 co-occurrences were observed for three transcription factors –NF-kappa B subunits p50 and p65 (referred to as NF-kappa B in following text), c-Rel and Evi-1. We further analyzed significance of the PG4 DNA co-occurrences with NF-kappa B, c-Rel and Evi-1 by analyzing association with the control motifs (Table 2). As noted previously for some of the SES, chi-square for co-occurrence of the three TFBS with both control-PG4 DNA and (GX)_17_-control was at least an order of magnitude lower than that observed for the PG4 DNA co-occurrence, and may therefore indicate preferred association of the TFBS with structure.

**Table 2:** PG4 DNA association with transcription factor binding sites

	Number of co-occurrence			Chi-square$		
	NF-kappa B	c-Rel	Evi-1	NF-kappa B	c-Rel	Evi-1
PG4 DNA	651	1103	715	164257	144995	41270
Control-PG4 DNA	37	100	98	1387	1889	1024
(GX)_17_ control	17	48	162	486	354	2094

$ Significance analysis in each case was done based on randomly expected co-occurrence (Methods).

The observed co-occurrence for each of the three factors (1103, 715 and 651 TFBS for c-Rel, Evi-1 and NF-kappa B respectively) appears to be few in number considering the genome-wide context and large number of hotspots. This suggests that only a fraction of the recombination events could be explained by TFBS association, which implicates alpha-hotspot activity in human. In the alpha-hotspot model, transcription factors associate with the recombination machinery to initiate/induce recombination. Though there is demonstrated evidence for alpha-hotspot activity in yeast [53], only putative α hotspots have been characterized in humans [48]. Perhaps interestingly, genome-wide estimate of number of hotspots (about 25000 to 50000) is somewhat close to the estimated number of genes in human, and is largely suggestive of alpha-hotspot activity in human, as noted in an earlier study [40]. On the other hand, in the same study it was observed that hotspots are enriched outside transcribed domains (peaking at a distance of ∼30 kb from genes in either direction). This precludes the presence of large number of transcription factors within hotspots and is consistent with our observation indicating relatively few instances of PG4 DNA-TFBS occurrences. Keeping in mind the fact that though few, these co-occurrences are not insignificant, it is tempting to speculate that such co-occurrences may account for some of the recombination activity. However, it must be considered with the caveat that our understanding of ‘transcribed region’ changes following more detailed analyses made possible by technological advances [54]. In this context, it is also important to note that evolutionary selection may have resulted in a skewed estimate of recombination and therefore these results are based on analysis of realized recombination regions rather than actual recombination.

Interestingly, NF-kappa B has been found to associate with a meiotic recombination hotspot present in the second intron of the mouse Eβ gene, where NF-kappa B's role in recombination has been implicated [55]. It is also interesting to note, in the context of our results that DNA binding subunits of NF-kappa B has been observed to bind to non-B DNA motifs [56]. These findings support our observations showing significant NF-kappa B-PG4 DNA co-occurrence within hotspots. Though our findings for the first time directly indicate the possibility of association of any human factor to PG4 DNA in the context of recombination, it is not unusual. Several meiosis-specific proteins in *S. cerevisiae* have been demonstrated to bind G4 DNA [34], [35], [44]. Recent findings strongly implicate non-B DNA conformations (such as triplexes, cruciform's, slipped structures, left-handed Z-DNA and G4 DNA) in initiation of chromosomal double strand breaks (DSB) leading to recombination-repair events and genomic rearrangements in general, causing several human diseases [1], [57] that resulted from gross deletions, inversions, duplications, translocations and also polymorphisms that were related to the structural motif and not sequence *per se* (reviewed in [1]). Notably, direct structure-specific interaction of the RAD51 accessory protein RAD51AP1 was reported in strand exchange during homologous recombination [6]. Interestingly, analysis of 222 breakpoints showed significant association of DSB with one or more non-B DNA structures [58] and a spontaneous translocation within the oncogene *bcl-2* has been reported to contain non-B DNA structures, which are cleaved by RAG proteins within the major breakpoint region [59]. Thus other than G4 DNA, non-B DNA conformations in general, and more importantly role of the structural form in such processes have gained support from recent findings.

In summary, we looked at the existence and relative enrichment of PG4 DNA within hotspots with the reasoning that any distinct role in recombination will show significantly different presence of PG4 DNA in hotspots with respect to coldspots in a genome-wide context. Our findings show enrichment of PG4 DNA within hotspots. Significant association with transcription factors, c-Rel, NF-kappa B subunits and Evi-1 noted by us is also interesting in the context of transcription factor-mediated recombination events. Based on these and other findings reported herein we predict a functional role of G4 DNA, either directly or indirectly, on interaction with other *trans*-factors, in recombination.

## Materials and Methods

### PG4 DNA searching, mapping and analysis within hotspots and flanking regions

PG4 DNA sequences were identified using scripts written in Perl. Nucleotide sequences were taken in FASTA format. Using the script we searched for the following motif G_[3–5]_N_[1–7]_G_[3–5]_N_[1–7]_G_[3–5]_N_[1–7]_G_[3–5]_ where G denotes guanine and N any nucleotide including G and number of nucleotides is given in the subscript. The algorithm used to identify PG4 DNA is conceptually similar to ones reported earlier [60] and has been described in detail before [8]. Briefly, using the above sequence pattern all PG4 DNA sequences were first identified. In case of overlapping motif sequences, which could result in more than one G4 DNA these were stitched together to produce tracts, where multiple PG4 DNA sequences are present and could result in more than one G4 motif. This tract information was used for analysis. We first found all PG4 DNA in a given stretch (hotspot/coldspot or flanking). For ease of presenting the data we calculate density per 100 bases. Motifs were always scored for the highest stem size, *i.e*, a motif with stem size of five once identified was not considered for any of the lower stem size. A control-PG4 DNA sequence (Text S1), which was very similar to the PG4 DNA motif but unlikely to adopt G4 DNA structure, was also used for analysis. This had the following sequence pattern: N_25_−G_3_−N_1–7_−G_3_−N_1–7_−G_3_−N_25_ with the following two restrictions: (i) two contiguous G's were not allowed at the 25 mer ends and (ii) two loops (underlined) were allowed to have all nucleotides but no two contiguous G's. Though the sequence pattern for G4 motif analyzed herein is most studied, few other sequence patterns can also result in G4 motifs (reviewed in [16], [61]). Furthermore, recent reports suggest single G bases can also contribute to the tetrad assembly [62]. Considering these, we used a second control motif, the (GX)_17_-control (where X = A/T/C but not G). This 34-mer control was of G-content and length such that it represents a PG4 DNA in sequence pattern but complete disruption of the G-tetrad arrangement renders it a highly unlikely candidate for G4 structure formation. We also noted in an earlier study Vorlickova and coworkers have shown that GC-repeats are reluctant to adopt tetraplex forms [63]. GA and GT repeats the other possibilities offered by the (GX)_17_-control are highly unlikely to adopt the G4 structure.

### Hotspots sequence retrieval and PG4 DNA analysis

We analyzed 25618 autosomal recombination hotspots from the 26177 regions reported by Myers *et al.* [40] (out of 26177 hotspots, 520 regions on X-chromosome were excluded and entire sequence information for 39 regions could not be retrieved from Build 35). Out of 9299 autosomal coldspots reported (http://mathgen.stats.ox.ac.uk/Recombination.html), 9290 coldspots could be retrieved and corresponding 9290 hotspots (matched for length and SNP density) were taken for analysis. Additionally, data on 32996 hotspots was obtained from the HapMap website for build 35 (http://hapmap.org/downloads/recombination/2006-10_rel21_phaseIII/). All coordinates were NCBI Human Genome build Version 35 and analysis was done primarily for the + strand. The − strand was analyzed for matched hotspots and coldspots. To analyze sequence flanking 32996 hotspots we obtained equal amount of region (for a given hotspot) both on the telomeric and centromeric sides of the hotspot. This would effectively give twice the length of the hotspot, therefore for comparison PG4 DNA density of the flanking regions was averaged. Regions of differing recombination potential were segregated from the HapMap data based on recombinogenicity unit cM/Mb for further analysis and designated as hotspots (≥10 cM/Mb) or coldspots (≤0.1 cM/Mb). These sequences were then used for analyzing the PG4 DNA content using in-house software. PG4 DNA density was expressed as number of nucleotides that would adopt structure divided by total sequence length of a given region. *R*_PG4/GC_ or PG4 DNA density normalized for GC content was obtained by dividing the PG4 DNA density by GC content of a given region; GC content was calculated using in-house software. GC% of each spot was determined and averaged across all hotspots or all coldspots. Average GC content (42.63%) in 25618 hotspots was higher than in 9290 coldspots (40.30%; p = 0.0007). Average GC% in case of the matched 9290 hotspots was 41.55% (versus 40.30% for the coldspots, p = 0.02). *R*_PG4/GC_ was calculated for each designated region independently and compared to check relative enrichment. Significance analysis (‘significant’ when mentioned in text implies ‘statistical significance’ unless otherwise specified) of difference in *R*_PG4/GC_ between regions of high and low recombination was done using the non-parametric statistical tests, Mann Whitney U- test (two un-related samples; in case of all hotspots and coldspots obtained from Myers et al.) or the Wilcoxon Signed Ranks Test (for two related samples; matched hotspots and coldspots/hotspots and flanking regions). Chromosome-wise average of *R*_PG4/GC_ was obtained to represent in figures.

### Analysis of short enriched sequences (SES)

6 to 9-mer sequences that were enriched with hotspots (SES), as reported in a previous study [40] were mapped within all hotspots and their co-occurrence with PG4 DNA observed using in-house software and the significance analyzed as given below. Seven SES that topped the list in reference 33 were selected for our study: GGGGGT, CCTCCCT, CCTCCCTG, CCCCACCCC, CCTCCTCT, CCACGTGG, TACTGTTC.

### Analysis of co-occurrence with transcription factor binding sites (TFBS)

Transcription factor binding sites were mapped within hotspots using the P-Match program available from TRANSFAC [52]. 32996 hotspots were analyzed by using scripts written in Perl, which submitted the sequence data to TRANSFAC and retrieved the TFBS in HTML format. This HTML file was further processed to obtain the corresponding TFBS for each hotspot. Then their co-occurrence with PG4's within a 50 bp window was found for each hotspot. Significance of the observed co-occurrence was analyzed after calculating the randomly expected co-occurrence for each factor using the method described below.

### Significance analysis for co-occurrence

To analyze the significance of co-occurrence of two elements either, PG4 DNA and SES or PG4 DNA and TFBS we first evaluated the randomly expected frequency of co-occurrence of any two elements given the individual frequency of their occurrence. The actual co-occurrence was then compared with random expectation of co-occurrence frequency to analyze significance. This is based on a previously published method [47]. Briefly, (F (f1, f2)) the frequency of co-occurrence of two factors f1 and f2 within m-base pairs (window size) in any n-base pair long sequence is given by 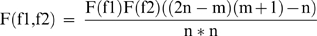
where F(f1) and F(f2) are independent frequencies of factors f1 and f2 in the n-base pair long sequence.

The actual co-ocurrence of two elements was determined and used to estimate significance by calculating
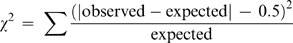
A degree of freedom = 1 was used to exclude false positives using a simple Bonferroni correction. For 32996 hotspots a significance level of p = 0.01/32996 corresponds to a chi-square of 26.23. Observed chi-square value for each co-occurrence is given in Table 1 and 2.

### DNase I hypersensitive sites analysis

DNase I hypersensitive sites (DHS) were obtained from report published by Sabo et al [43] . We mapped DHS within hotspots and coldspots reported by Myers et al [40]. All DHS were observed to be completely inside the hotspots or coldspots, in other words we did not see any partial overlap between DHS and hot or coldspots. About 1.05% of hotspots and 0.35% of coldspots overlapped with the DHS reported by Sabo et al [43].

## Supporting Information

Text S1 (0.03 MB DOC)

Table S1 (0.03 MB DOC)

Table S2 (0.03 MB DOC)

Table S3 (0.03 MB DOC)
