# Supplementary material for: Genome-Wide Analyses of Recombination Prone Regions Predict Role of DNA Structural Motif in Recombination
Source: PLoS One. 2009 Feb 9;4(2):e4399. doi: 10.1371/journal.pone.0004399 (PMC2635932; doi:10.1371/journal.pone.0004399)
Supplement: Text S1 — (0.03 MB DOC) [file pone.0004399.s001.doc]

**Supplementary Information**

# Genome-Wide Analyses of Recombination Prone Regions Predict Role of DNA Structural Motif in Recombination

Mani et al.

G. N. Ramachandran Knowledge Centre for Genome Informatics, Proteomics and Structural Biology Unit, Functional Genomics Unit, Institute of Genomics and Integrative Biology, CSIR, Mall Road, Delhi 110 007, India

**Analysis of control motifs**

The restricted nature of the control motifs give overall lower number of occurrences. Therefore we also analyzed a control motif where the adjacent length was limited to 10-mers (N-10 control motif) on both sides (Table S1). This motif gives relatively more number of occurrences and shows averaged density in hotspots and coldspots is insignificant. However, one must note that the N-10 control can result in G4 motifs with a loop size more than 10 bases.
