# Supplementary material for: Genome-Wide Analyses of Recombination Prone Regions Predict Role of DNA Structural Motif in Recombination
Source: PLoS One. 2009 Feb 9;4(2):e4399. doi: 10.1371/journal.pone.0004399 (PMC2635932; doi:10.1371/journal.pone.0004399)
Supplement: Table S1 — (0.03 MB DOC) [file pone.0004399.s002.doc]

**Table S1.** Details of occurrence and significance for PG4 DNA and control motifs compared for hotspots and coldspots

|  | # of hotspot/coldspots with at least one PG4/control motif (Myers *et al.*) | | p-value for comparison of average *R*_PG4/GC_ in hotspots vs. coldspots |
| --- | --- | --- | --- |
|  | 9290 matched*^a^* hotspots | 9290 coldspots | 9290 matched hotspots/coldspots |
| PG4 DNA | 1529 | 1285 | 1.6 x 10^-6^ |
| Control, n = 25*^b^* | 191 | 189 | 0.8 |
| Control, n = 10*^b^* | 1031 | 902 | 0.7 |
| Control,  (GX)_17_ | 281 | 276 | 0.6 |

*^a^* 9290 hotspots and coldspots matched for length and SNP density [1] . *^b^* n as defined in the pattern N_n_ - G_3_ - N_1-7_ - G_3_ - N_1-7_ - G_3_ – N_n_; this gives an adjacent length of 25 or 10-mer with restrictions as mentioned in Methods.
