# Supplementary material for: Genome-Wide Analyses of Recombination Prone Regions Predict Role of DNA Structural Motif in Recombination
Source: PLoS One. 2009 Feb 9;4(2):e4399. doi: 10.1371/journal.pone.0004399 (PMC2635932; doi:10.1371/journal.pone.0004399)
Supplement: Table S2 — (0.03 MB DOC) [file pone.0004399.s003.doc]

**Supplementary Table S2. PG4 DNA association with short enriched sequences (SES)**

| **short enriched sequences (SES)** | **Observed number of co-occurrence** | | |
| --- | --- | --- | --- |
|  | PG4 DNA | Control-PG4 DNA | (GX)17-control |
| **CCCCACCCC** | 106 | 6 | 2 |
| **CCTCCTCT** | 88 | 7 | 4 |
| **CCACGTGG** | 65 | 2 | 1 |
| **TACTGTTC** | 15 | 2 | 5 |
