# Supplementary material for: Genome-Wide Analyses of Recombination Prone Regions Predict Role of DNA Structural Motif in Recombination
Source: PLoS One. 2009 Feb 9;4(2):e4399. doi: 10.1371/journal.pone.0004399 (PMC2635932; doi:10.1371/journal.pone.0004399)
Supplement: Table S3 — (0.03 MB DOC) [file pone.0004399.s004.doc]

**Supplementary Table S3.** PG4 DNA association with 10 transcription factor binding sites

| **Transcription Factor** | **No. of co-occurrence** | **Chi-square^$^** |
| --- | --- | --- |
| **c-Rel** | 1103 | 144995.4 |
| **Evi-1** | 715 | 41270.5 |
| **NF-kappa B** | 651 | 164256.8 |
| **E2F** | 204 | 77813.2 |
| **E2** | 194 | 63131.05 |
| **Nkx2-5** | 161 | 7203.8 |
| **NRF-2** | 159 | 66777.36 |
| **STATx** | 145 | 17876.63 |
| **Elk-1** | 119 | 20466.73 |
| **RREB-1** | 103 | 93476.6 |

^$^ Significance analysis in each case was done based on randomly expected co-occurrence (see Methods).
